# Gingival crevicular fluid MMP-8-concentrations in patients after acute myocardial infarction

**DOI:** 10.1186/1746-160X-7-1

**Published:** 2011-01-10

**Authors:** Vicky Ehlers, Ines Willershausen, Joachim Kraft, Thomas Münzel, Brita Willershausen

**Affiliations:** 1 Department of Operative Dentistry, University Medical Centre of the Johannes Gutenberg University Mainz, Germany; 2 Institute for Dental Material Sciences and Technology, University Medical Centre of the Johannes Gutenberg University Mainz, Germany; 3 Department of Cardiology and Angiology, University Medical Centre of the Johannes Gutenberg University Mainz, Germany

## Abstract

**Background:**

The aim of this study was to determine the presence of matrix metalloproteinase-8 in the gingival crevicular fluid (GCF) of patients after acute myocardial infarction (AMI).

**Methods:**

A total of 48 GCF samples from 20 AMI patients, hospitalized at the Department of Cardiology and Angiology of the Johannes Gutenberg University Mainz, were investigated. Besides the myocardial infarction all patients suffered from chronic periodontal disease. Fifty-one GCF samples from 20 healthy age matched individuals with similar periodontal conditions served as controls. The dental examination included the assessment of oral hygiene, gingival inflammation, probing pocket depth, clinical attachment level and X-ray examination. The study was only carried out after the positive consent of the regional ethic commission. A quantitative assessment of aMMP-8 levels in the gingival crevicular fluid was performed with the help of the DentoAnalyzer (Dentognostics GmbH, Jena, Germany), utilising an immunological procedure.

**Results:**

The aMMP-8 concentrations found in the gingival crevicular fluid of the AMI patients significantly differed (p = 0.001; mean value 30.33 ± 41.99 ng/ml aMMP-8) from the control group (mean value 10.0 ± 10.7 ng/ml aMMP-8). These findings suggest that periodontal inflammation in AMI patients might be associated with higher MMP-8-values compared to the healthy controls.

**Conclusions:**

The acute myocardial infarction seems to influence the degree of periodontal inflammation, thus the measurement of the gingival crevicular fluid MMP8 levels seems to be a helpful biochemical test to obtain information about the severity of the periodontal disease.

## Background

Clinical and radiological diagnoses as well as microbial tests are the basis for an effective periodontal therapy. The severity of periodontitis can be further quantified by the presence of inflammatory parameters i.e MMPs in the gingival crevicular fluid. This knowledge is helpful for clinicians in finding the most effective treatment modality [1].

Matrix metalloproteinases (MMPs) are a family of enzymes that degrade various components of the extracellular matrix [2,3]. Increased expression and activity of MMPs has been linked with rheumatoid arthritis, tissue degradation, bone resorption [4,5], tumor metastasis and ischemic injury [6]; MMP-9 for instance is physiologically found in the heart and its up regulation is associated with heart failure, indicating a possible role of MMP-9 in cardiomyopathy [7]. In the gingival crevicular fluid (GCF) MMP-8 is regarded as the most prominent collagenase (collagenase-2) associated with the breakdown of connective tissue and periodontal progression [8-11]. It is therefore regarded as a helpful variable in diagnostic research [12]. Periodontal treatment concepts such as scaling and root planning are capable of decreasing the MMP8 levels in the gingival crevicular fluid [11]. However, when permanently exposed to high MMP8 concentrations, inflamed periodontal pockets are a risk of irreversible tissue destruction.

According to the World Health Organization Statistics coronary heart disease (CHD) is the principal cause for death and responsible for 50% of all death in the western industrialised countries [13]. It has also been reported that patients with CHD have poorer dental health than controls without CHD [14,15] with periodontitis associated with the development of artherosclerosis.

Beck et al. [16] postulated that periodontal disease and artherosclerosis seemed to share a similar pathway and that certain individuals might be more likely than others to respond to higher levels of inflammatory stimuli. These factors lead to excessive production of cytokines and other inflammatory mediators, enhancing the development periodontal and arterial cell wall lesions. The matrix metalloproteinases are among such inflammatory mediators. Up regulation of MMP-8 for weeks or even months is correlated with inflammation and bone resorption [8,9,11]. In healthy individuals exists a balance between matrixmetalloproteinases (MMPs) and tissue inhibitors of matrixmetalloproteinases (TIMPs). The inflammation triggers an immunological response, which immediately leads to the activation of matrixmetalloproteinase-8 and to the reduction of collagen [17]. Thus, the disturbed balance of MMPs and TIMPs through excessively unregulated MMP-8 has to be diagnosed at an early stage, even before the clinical signs are evident, in order to prevent severe periodontitis.

The present study was performed to evaluate the level of active matrix metalloproteinase-8 (aMMP-8) in the GCF from patients after acute myocardial infarction and from healthy controls and to compare the concentration of aMMP-8 between these groups. Moreover, their clinical periodontal status - including gingival inflammation, probing pocket depth, clinical attachment level, recession and dental hygiene - was assessed.

The aim of this study was to determine a possible elevation of aMMP-8 concentrations in AMI patients, indicating that therapies to decrease MMP-8 activity may be beneficial to prevent further or severe periodontal breakdown and bone resorption particularly in risk patients.

## Methods

### Study Population

In the study a total of 48 gingival crevicular fluid (GCF) samples from 20 patients (42 to 84 years) with acute myocardial infarction were collected and analyzed. The samples were collected within one week after the patients had experienced the myocardial infarction. These samples were compared to 51 GCF samples from 20 healthy age matched controls from outpatients of the Dental University of Mainz. Both AMI patients and control patients showed clinical and radiological signs of moderate chronic periodontal diseases (PD ≤ 5 mm).

All subjects included in this examination gave informed consent to participate and the study was approved by the institutional review board of the University of Mainz, the ethics committee and the National Board for Radiation Protection.

Inclusion criteria were the presence of an acute myocardial infarction verified by characteristic electrocardiogram changes and evaluation of serum enzymes (serum glutamic oxaloacetic transaminase, creatinine phosphokinase). All patients had suffered a recent history of acute myocardial infarction as verified by hospitalization at the Department of Cardiology and Angiology of the University of Mainz. The majority of the hospitalized AMI collective either suffered from chronic periodontitis or were edentulous. Only those patients with moderate chronic periodontis were included in the present study. The dental examination and assessment of sulcular fluid samples aMMP-8 was carried out directly bed-side at the Department of Cardiology and Angiology and only patients deemed clinically stable to undergo a thorough dental examination and measurement of aMMP-8 were included in the study.

All control patients also suffered from moderate chronic periodontitis but were in good general health with exclusion criteria being a history of cardiovascular disease, hypercholesterolemia or any other severe illness. The control patients were not in systemic periodontal therapy within the last 6 months.

### Oral examination

For this investigation only AMI patients and volunteers with more than five teeth could be included.

The selection of a total of 5 teeth per mouth as minimum inclusion criteria resulted in the observation that a great number of AMI patients were edentulous or showed a low number of teeth. Therefore a third of the recruited patients had to be excluded from the study.

The control patients were examined during a clinic visit. All subjects underwent a questionnaire, a radiological investigation and a thorough oral examination. The dental examinations included standard periodontal parameters probing pocket depths (PD), clinical attachment level (CAL), recession. Moreover, oral hygiene (PI, *Quigley-Hein-Plaque-Index*) [18] and bleeding index (PBI, *Saxer/Mühlemann*) [19] were recorded by the attending dentist. All measurements were recorded at six sites on each tooth and a total of 2-4 teeth per patient were analysed, using a standard periodontal probe (PCP 15, Hu-Friedy, Chicago, IL, USA).

### Measurement of aMMP-8

GCF was collected from up to four teeth before any treatment measures from AMI patients and the control group using a standardized MMP-8 collection strips [12]. All patients showed a moderate chronic periodontal disease (PD ≤ 5 mm) but no symptoms of acute aggressive periodontal disease. The evaluated teeth included anterior and posterior teeth. The aMMP-8 collection (aMMP-8 levels: healthy conditions: 0-7 ng/ml; severe periodontitis > 65 ng/ml) [8,17,20,21] was carried out with the help of paper strips, which were placed in the periodontal pockets for 30 seconds [1]. The aMMP-8 was immediately eluted from the strips for 30 seconds and quantitatively assessed with the *DentoAnalyzer *(Dentognostics GmbH, Jena, Germany) [20,21]. The *DentoAnalyzer *automatically conducted the entire assay process and the result of the test was revealed within 12 minutes chair-side. With the help of this assay the concentration of aMMP-8 can be measured in a range from 2 ng/ml aMMP-8 eluate up to 200 ng/ml aMMP-8 eluate [22-25].

### Medical history

At the time of enrollment age and gender were assessed for all patients. All subjects were also asked for classical cardiovascular risk factors including family history of coronary artery disease, smoking and diabetes mellitus.

The patients diagnosed with an acute myocardial infarction had a medical history of an ST-elevated myocardial infarction (STEMI) or Non-Stemi cardiac events (NSTEMI). All of the AMI patients were hospitalized and blood samples were taken as a means of clinical diagnosis.

The blood pressure (hypertension was defined according to WHO guidelines), the serum values for glucose, hemoglobin, glycosylated hemoglobin (HbA1c), total cholesterol, triglyceride (TG), leukocytes, fibrinogen, the albumin excretion rate (AER), creatinine phosphatase and CRP serum levels were obtained from the patients during hospitalization.

### Statistical analysis

All statistical analyses were performed using statistical software (SPSS, 15.0 for Windows, Chicago, IL, USA). Mean and standard deviation (SD) were calculated for compare the data gained from the acute myocardial patients and the control subjects. For the statistical evaluation of the differences between the two patient groups, the Chi-Quadrat test and the Mann-Whitney-test were used. In all test procedures a significance level of p < 0.05 was considered statistically significant.

## Results

All patients who were hospitalized on account of an acute myocardial infarction (AMI) also suffered from moderate chronic periodontitis. They had a mean age of 64.5 years (SD: 12.7 years), and the majority were of male gender (85.4% male, 14.6% female). The age (63 years; SD: 6.1 years) and gender (male (60.8%) and female (39.2%)) matched controls also suffered from moderate chronic periodontitis, but showed no sign of cardiovascular disease. Statistically there were no odd differences between both age and gender of the patients and controls.

The concentrations of aMMP-8 were significantly elevated in gingival crevicular fluid (GCF) samples from patients after acute myocardial infarction (p = 0.001; median 13.5, mean value 30.33 ± 41.99 ng/ml aMMP-8 eluate) when compared to the GCF samples from the control group (median 6, mean value 10.02 ± 10.7 ng/ml aMMP-8 eluate) (Table 1).

**Table 1:** Clinical data of the AMI patients and the controls.

Patients	Plaque-Index (%)					MMP8 (ng/ml) ± SD
	**grade** **0**	**grade** **1**	**grade** **2**	**grade** **3**	**grade** **4**	

AMI	16.7	18.8	35.4	27.1	2.1	30.3 ± 41.9

Control	33.3	39.2	9.8	13.7	3.9	10.0 ± 10.7

Concerning the periodontal parameters such as pocket depths, recessions and clinical attachment levels there were no statistically odd differences regarding the periodontal sites from AMI patients and from controls. However the AMI patients demonstrated poorer dental hygiene than the control patients and the Plaque-Index (PI) was significantly higher (p = 0.002). In the AMI patient group, periodontal sites grade 2 (35.4%) and grade 3 (27.1%) dominated, 18.8% had grade 1, 16.7% grade 0 and 2.1% grade 4. Grade 5 was recorded neither among the AMI patients nor among the controls. As expected grade 1 (39.2%) and grade 0 (33.3%) were predominant in the control group, followed by grade 3 (13.7%), grade 2 (9.8%) and grade 4 (3.9%) (Table 1).

The degree of gingival inflammation represented by PBI showed no odd differences between periodontal sites of AMI patients and healthy patients (p = 0.775). There were also no correlations between aMMP-8 concentration and gingival recession or clinical attachment level. A significant association was found between aMMP-8 concentration and pocket depth both among the AMI patients and among the controls (p = 0.002).

**Figure 1:**
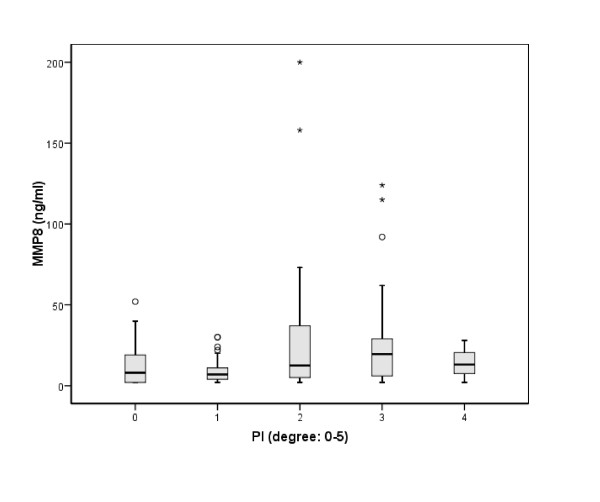
**Significant correlation between MMP-8-concentration and Plaque Index (PI) in the present study population**.

MMP-8 concentrations and Plaque-Index revealed a statistically significant correlation considering both groups together (patients and controls) (p = 0.027) (Figure 1). There was also a week connection between MMP-8 concentrations and gingival inflammation in both groups (p = 0.883), but this was not statistically significant.

Moreover, within the AMI patient group there were no statistically differences between MMP-8 values and stemi/non stemi or between MMP-8 concentrations and smoking habits. No statistically odd correlations were found between medical data like blood glucose concentrations, number of leukocytes or LDL-values and MMP-8 concentrations.

## Discussion

In this study changes in gingival crevicular fluid MMP-8 levels in patients after myocardial infarction were investigated. We found significantly higher MMP-8 concentrations in myocardial infarct patients compared to patients without cardiovascular disease but with a similar periodontal condition. The importance of the collagenase activity in GCF, represented mainly through MMP-8, as an indicator for periodontal collagen breakdown and alveolar bone resorption could be demonstrated in a group of postmenopausal women during a 2-year follow up study [26].

Numerous studies have demonstrated a correlation between CHD and periodontal disease [16,27,28]. In a systematic review Humhrey et al. [29] showed that periodontal disease including gingivitis, bone loss and missing teeth is an independent but relatively weak risk factor for CHD. Individuals suffering from periodontal disease have approximately a 24-35% increased risk of developing CHD. In our study the AMI patients showed higher MMP-8 levels, which might represent, together with the present periodontal disease, a further negative influence on the progression or severity of the myocardial infarction.

It has been demonstrated that both cardiovascular disease (CVD) and periodontal disease are more likely to occur in male, older, smoking patients, suffering from diabetes. Furthermore a low socio-economic status, stress and social isolation are made responsible for the occurrence of CVD [27,30]. In the study of Furuholm et al. [31] the salivary MMP-8 levels in patients with CHD were measured and compared with healthy control persons. The authors found that the CHD patients showed significantly higher levels of MMP-8. This suggests that periodontal disease and CHD might have similar causative pathways.

In the Consensus Report of the Sixth European Workshop on Periodontology Kinane et al. [32] found evidence a periodontal disease contributes to the total infectious and inflammation burden and may contribute to cardiovascular events and stroke in susceptible subjects. They report that the impact of periodontal therapy should investigated further.

Different studies evaluated to what extent chronic infections are involved in the pathogenesis of CHD and how chronic infections of dental origin are cumulative in patients with CHD or acute myocardial infarction [33-35]. In the study of Slade et al. [36] the relationship between periodontal disease, C-reactive protein and the risk of artherosclerosis was examined among adults. A moderately elevated serum C-reactive protein (CRP) concentration is regarded as a marker of inflammation and a risk factor for CVD.

Unrecognized infections such as periodontal disease may induce acute-phase response, elevating CRP levels. Mean CRP level among people with extensive periodontal pockets was one-third greater than that for people with less extensive periodontal pockets. They concluded that extensive periodontal disease and body mass index (BMI) are associated with increased CRP levels in otherwise healthy, middle-aged adults. Therefore the authors suggested the need for medical and dental diagnoses when evaluating sources of acute-phase response in some patients [36]. Grau et al. [37] investigated whether periodontal disease, including periodontitis and gingivitis, is a risk factor for cerebral ischemia. They found that periodontal disease is an independent risk factor for cerebral ischemia in men and younger subjects.

In the present study except for the Plaque-Index (PI) all other periodontal parameters like probing depth, recession, clinical attachment level and gingival inflammation were not statistically different from the controls. The statistically significant elevation of plaque accumulation could be explained by the poorer dental hygiene of the hospitalized AMI patients. Other studies have shown that patients after AMI had a more insufficient dental index than control persons [38]. Moreover the positive correlation between PI and MMP-8 levels might be caused by the potential inflammatory effect through the dental plaque. In a large epidemiological survey Mattila [39] showed that chronic periodontal diseases are associated with an increased risk of CHD and that the number of missing teeth has been linked with elevated risk levels for CHD. In the Kuopio Ischemic Heart Disease Risk Factor (KIHD) study of Tuomainen et al. [40] baseline serum levels of MMP-8 in a great number of Finnish men were measured and re-examined 4 years later. They found that serum MMP-8 concentrations were significantly higher in men with CVD than in men without signs of CVD. These data imply that MMP-8 levels are elevated in prevalent or subclinical atherosclerosis.

Therefore it is important that patients with CVD, CHD or hospitalized AMI patients have an early dental appointment to control and treat the periodontal disease. The aim of this early treatment is to eliminate or reduce high aMMP-8 activity with the help of periodontal therapy and to prevent further inflammation and periodontal destruction. Several studies postulated that patients who have periodontitis have a higher risk of future CVD [33,41]. De Stefano et al. [41] found that people with periodontitis had a 25 percent increased risk of suffering from CVD. Moreover they found that men with periodontitis had a 1.72 relative risk compared with men without periodontitis.

In this study statistically significant correlations were found between gingival crevicular MMP-8 concentrations and periodontal parameters.

To the best of our knowledge, there are currently no further studies with MMP-8 and patients after AMI. Another study related to MMPs was found, where Romanic et al. [42] investigated the role of MMP-9 in myocardial infarction utilising an MMP-9 knockout mouse model. Their results suggested that MMP-9 plays an important role in ischemia-reperfusion-induced myocardial infarction and that MMP-9 could be a target for prevention or treatment of acute ischemic myocardial injury.

## Conclusions

It has been reported that chronic periodontal disease is a weak risk factor for coronary heart disease. The early diagnostic and information about the severity of this inflammatory disease with the help of special biochemical tests is very helpful particularly for patients after myocardial infarction.

Thus, our results concerning the measurement of the MMP-8 levels in gingival crevicular fluid give information about a possible risk of the progression of periodontal disease. Consequently this marker plays an important role in the diagnosis of the severity of periodontal disease which is might be beneficial for patients after myocardial infarction. On the one hand it may be useful to treat patients with periodontitis in order to minimize one of the possible cardiovascular risk factors and, on the other hand, to improve dental hygiene among AMI patients. This can be considered helpful to prevent the progression of the periodontal disease and to minimize a possible interaction between the periodontal and the cardiovascular disease.

## Competing interests

The authors declare that they have no competing interests.

## Authors' contributions

VE and IW carried out the study and examined all patients. VE and IW performed the statistical analysis. TM and JK participated in the design of the study. BW conceived of the study, and participated in its design and coordination. All authors read and approved the final manuscript.
